# Six years of One Health action in Africa: evaluating outcomes, mechanisms, and lessons from OHRECA (2020–2025)

**DOI:** 10.1016/j.soh.2026.100166

**Published:** 2026-07-24

**Authors:** Steven Lâm, Bernard Bett, Arshnee Moodley, Florence Mutua, Lian Thomas, Delia Grace

**Affiliations:** a Health Program, International Livestock Research Institute, Nairobi 00100, Kenya; b Department of Health Sciences, Wilfrid Laurier University, Waterloo N2L 3C5, Canada; c Department of Veterinary and Animal Sciences, University of Copenhagen, Frederiksberg C 1958, Denmark; d Royal (Dick) School of Veterinary Studies, University of Edinburgh, Edinburgh EH25 9RG, United Kingdom; e Natural Resources Institute, University of Greenwich, Greenwich ME4 4TB, United Kingdom

**Keywords:** One Health, Research-for-development, Capacity building, Program evaluation, Africa

## Abstract

**Background:**

There is growing momentum towards using One Health approaches to address health threats emerging when human activities come in close contact with animals and shared environments. In Africa, where the burden of such threats is disproportionately high, strengthening One Health capacity is important for improving health system preparedness. Although substantial investments have been made in One Health capacity building across low- and middle-income countries, there remains limited evidence on how such initiatives achieve sustainable outcomes and the conditions that enable their success.

**Case description:**

The One Health Research, Education, and Outreach Centre in Africa (OHRECA), a six-year research-for-development initiative (2020–2025) implemented across multiple African countries, was evaluated. Using a realist-informed mixed-methods evaluation, this study explores how and why OHRECA achieved its outcomes.

**Key outcomes:**

Findings suggest that OHRECA contributed to scientific productivity, workforce development, and more in the target countries. Learning-by-doing and partnerships emerged as key mechanisms while long time-investments and institutional continuity were important contextual conditions supporting these outcomes.

**Conclusions:**

Given the absence of baseline and comparator data for many outcomes, the findings should be interpreted as explanatory case study evidence rather than as a causal effectiveness assessment. Nevertheless, they offer transferable lessons for designing One Health capacity building efforts in African research systems and other low- and middle-income settings.

## Graphical abstract

**Figure:**
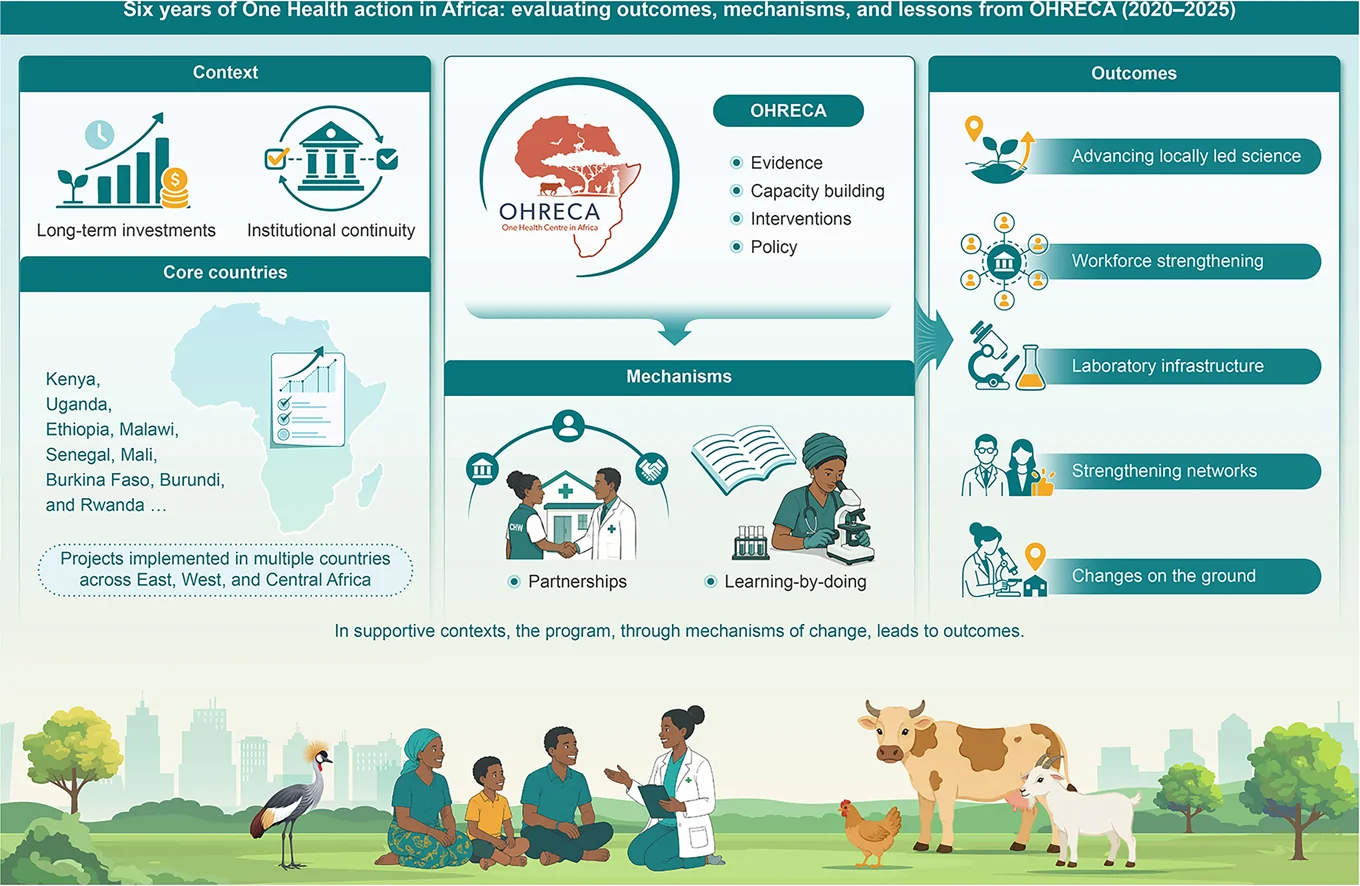

## 1 Background

Africa experiences the world's greatest burden of zoonotic diseases [1], bacterial antimicrobial resistance [2], and foodborne illnesses [3], many of which arise where human activity disrupts animal populations and shared ecosystems. The One Health approach recognizes that the health of humans, animals, and the environment are deeply interconnected, and therefore advocates for multisectoral collaboration to address health risks [4]. One Health has taken strong root across Africa with many strong networks, programs, and commitments [5,6].

Taking a One Health approach requires a critical mass of local scientists equipped in One Health competencies [7]. They are best placed to develop effective solutions given their understanding of local systems, cultures, and constraints. Furthermore, building local research capacity is important not only for achieving impact but also for ensuring long-term sustainability, as trained scientists are more likely to advance One Health initiatives in their own country. Yet, in under-resourced areas, a shortage of trained One Health professionals persists [8].

With growing demand for One Health capacity, the One Health Research, Education, and Outreach Centre in Africa (OHRECA) was established, building off earlier capacity strengthening efforts such as Afrique One and African One Health University Network [9,10]. To inform One Health workforce development efforts, this article aims to examine the center's achievements, including how and why outcomes occurred. To do so, it draws on qualitative data to examine underlying mechanisms and contextual influences, complemented by quantitative indicators that describe the scale and reach of these outcomes. In doing so, the study contributes evidence on factors that support effective One Health capacity strengthening initiatives. Further details are provided in the final evaluation report [11].

## 2 Methodology

The evaluation applied a mixed-methods approach, drawing on both qualitative and quantitative data. A realist evaluation framework guided the qualitative component, focusing on how outcomes were achieved, in what contexts, and why, through exploring the interactions between context, mechanisms, and outcomes [12]. This approach is well-suited to complex, multi-sectoral initiatives such as OHRECA.

Semi-structured interviews were conducted between July and September 2025—during the 6th and final year of the initiative—via Microsoft Teams or phone call. The interview guide was informed by realist principles and pilot tested and revised. Participants were purposively selected by the team based on gender, stakeholder type, and project involvement. Given the focus on understanding how the initiative was experienced across different stakeholder groups, sampling prioritized diversity of key perspectives rather than achieving theoretical saturation. Interviews were conducted in English and varied in duration from 30 to 90 min. Interviews were not audio-recorded or transcribed. Instead, detailed notes were taken during each interview and expanded immediately afterward to capture key points and observations relevant to the evaluation questions. This approach was considered appropriate for the evaluation's explanatory objectives. Because note-based documentation may have limited the level of detail available for analysis, participant review of interview summaries was used to verify and enhance the accuracy of the information captured.

We conducted thematic analysis to identify patterns or themes in the data [13]. Specifically, we applied several sequential phases of deductive coding, with results from one phase informing the focus on coding in the subsequent phase. In phase 1, we examined outcomes of the OHRECA initiative. Building from these findings, phase 2 focused on characterizing the mechanisms and contextual factors, as well as linking them to outcomes via C-M-O configurations (Table S1). Finally, in phase 3, we explored the challenges and lessons.

The lead author conducted all interviews and the initial coding. There was no formal double coding. While regular discussions among the authorship team were held to review the coding process, challenge interpretations, and refine themes, alternative interpretations of the data remain possible. These discussions involved authors with institutional knowledge of OHRECA, who provided contextual insight into program activities and implementation processes, as well as the external evaluator (lead author), who was familiar with International Livestock Research Institute (ILRI) projects but not directly involved in OHRECA and contributed an independent perspective. This process helped ensure that interpretations were grounded in the data while accounting for the broader program context. NVivo© software (v15) facilitated coding.

For the quantitative component, we examined OHRECA publications and citation metrics to assess scale and reach of research outputs. Publications were identified through CGSpace (the Consultative Group on International Agricultural Research [CGIAR] institutional repository, available at https://cgspace.cgiar.org/home) using the search term “OHRECA.” The search was conducted on 20 August 2025. The search returned all OHRECA outputs available in CGSpace, including peer-reviewed journal articles, reports, policy briefs, presentations, and videos. For the purposes of this manuscript, only peer-reviewed journal articles were included.

Bibliographic information for all eligible publications was extracted from CGSpace. Citation counts were obtained from Dimensions badges associated with each publication record, and Altmetric scores were obtained from the Altmetric data displayed within CGSpace records. The search identified 105 records. Following the exclusion of 30 records based on publication type and 8 duplicate records, 67 peer-reviewed publications were included for analysis, all of which acknowledge OHERCA funding.

Of note, many outcomes of interest were derived from open-ended qualitative data. As this evaluation focused on understanding how and why outcomes were achieved, rather than assessing effectiveness, it did not rely on baseline or comparator data. Evidence supporting reported outcomes was drawn from multiple sources, including participant interviews, program monitoring records, and bibliometric analyses. Findings were triangulated across these data sources where possible to strengthen the credibility of interpretations and to corroborate reported outcomes. Where available, quantitative indicators were used to describe the scale and reach of outputs and early outcomes (e.g. individuals trained, publications produced), complementing the qualitative analysis.

## 3 Case description

OHRECA (2020–2025; EUR 15 million) was launched just before COVID-19, a zoonotic disease that disrupted the world, led to significant losses of life, and crippled the global economy. COVID-19 was a reminder of the need to work holistically across multiple sectors as our health is connected to that of animals and our shared environment.

**Fig. 1:**
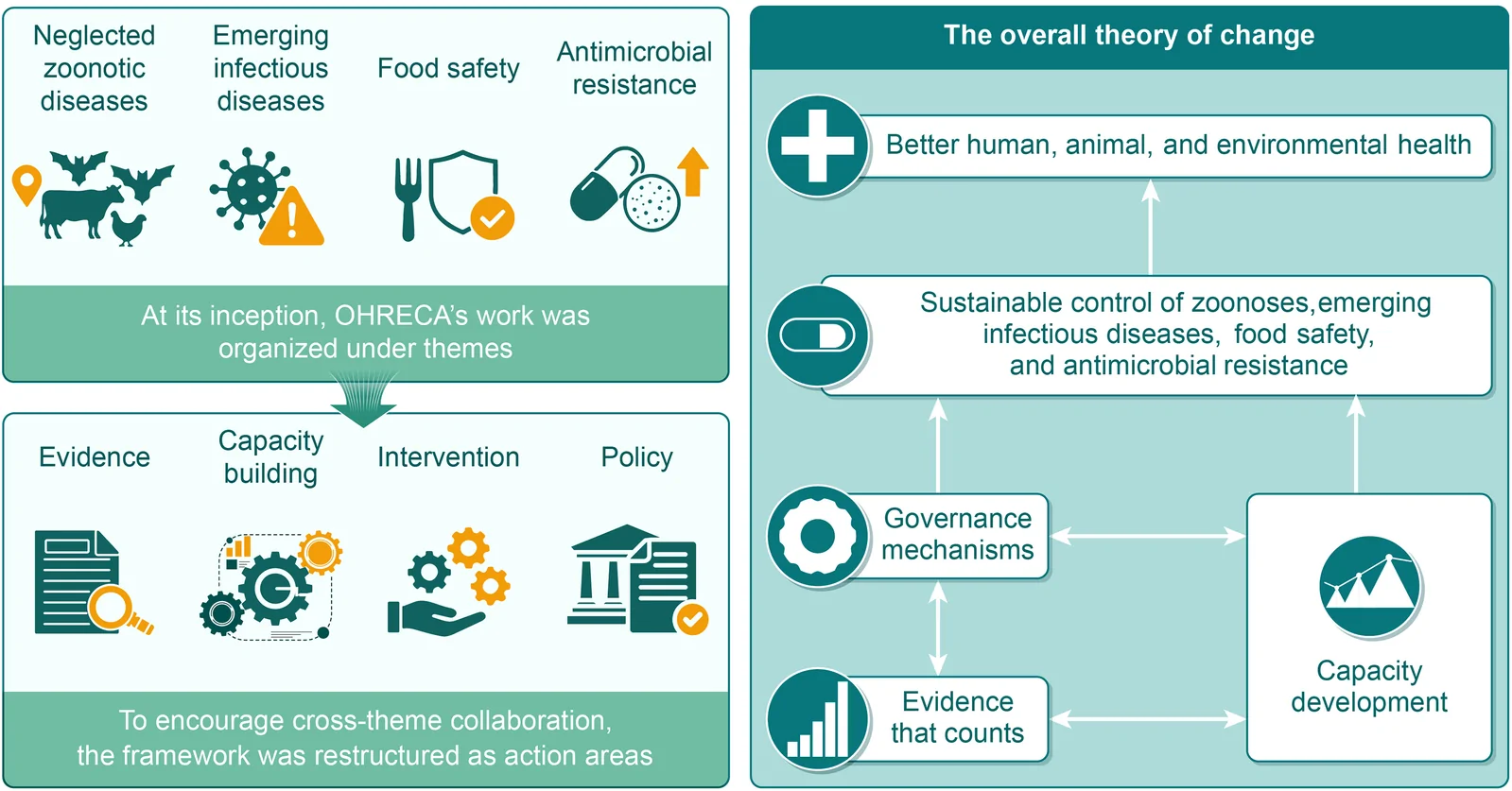
Re-structuring of OHRECA from theme-based activities to cross-cutting action areas in 2023. While the organization changed to improve integration, the overall Theory of Change (green boxes) remained consistent. Arrows indicate the intended pathways through which activities contribute to outcomes. Abbreviations: OHRECA, One Health Research, Education, and Outreach Centre in Africa; AMR, antimicrobial resistance.

At its inception, OHRECA's work was organized under four thematic areas: neglected zoonoses, emerging infectious diseases, food safety, and antimicrobial resistance (AMR), each supported by theme leads, including three veterinary epidemiologists and a microbiologist. A 10-member advisory committee comprising representatives from One Health institutions in Africa, Europe, and North America was later created to guide OHRECA. One of the committee's recommendations, for example, was to re-structure OHRECA in 2023 around four integrated themes (evidence, capacity building, intervention, policy) to encourage integrated research approaches (Fig. 1).

OHRECA aimed to generate new knowledge and build capacity to strengthen One Health policies and practice in Africa. Its primary approach was engaging graduate fellows in research-for-development, where research is as a tool to address real-world health and development challenges. In addition, OHRECA collaborated closely with implementation partners and One Health platforms to ensure that efforts were aligned with practice and policy needs. The initiative operated primarily in Kenya, Uganda, Ethiopia, Malawi, Senegal, Mali, Burkina Faso, Burundi, and Rwanda. Projects were informed by engagements across the region to understand priorities as well as strategic country selection to promote cross-country learnings (Table 1). Hosted at the ILRI, OHRECA leveraged ILRI's biosciences, environmental, and genomics facilities, as well as five decades of institutional experience in Africa [14] and its more recent leadership in One Health [15].

**Table 1:** OHRECA projects and their focal countries.

Action area	Title	Country
Evidence	Epidemiological studies for *Taenia solium*	Uganda, Kenya
	Tool development, dog population dynamics, and costing studies for rabies	Kenya
	Risk mapping, genomic analysis, and gender dynamics for Rift Valley fever	Kenya
	Risk mapping and molecular analysis for Crimean-Congo hemorrhagic fever	Burkina Faso, Kenya
	Risk analysis and intervention entry points for foodborne disease	Kenya, Burundi, Ethiopia, Rwanda
	Fate of antimicrobial residues in manure and cropping soils and links to greenhouse gas emission and environmental ecotoxicity	Kenya
	Behavioral and epidemiological research on drivers of antimicrobial use in humans and animals	Malawi
Capacity building	Long-term capacity building of graduate fellows	Sub-Saharan Africa wide
	Training of cohorts of veterinary students	Malawi
	Training curricula development for veterinarians on antimicrobial use	Uganda, Kenya
	Training national One Health platforms	Burkina Faso, Mali, Senegal, Côte d’Ivoire
	Training local One Health platforms	Kenya
Interventions	Mass vaccination campaigns for rabies	Kenya
	Zoonotic disease surveillance strengthening	Kenya
	Meat vendor training in informal markets	Kenya
Policy	Review of policies on antimicrobial resistance stewardship	Kenya, Malawi
	Review of Rift Valley fever policies	Kenya
	Engagement in policy dialogues	Sub-Saharan Africa wide

OHRECA provides a strong case study for understanding how and why outcomes are achieved within complex One Health initiatives. Using a realist evaluation approach, this study identifies mechanisms and conditions for impact, contributing to the growing evidence base on strategies for operationalizing One Health [16,17].

## 4 Results and discussion

A total of 20 OHRECA participants were interviewed (11 men and 9 women). Participants were primarily scientists (*n* = 9), followed by graduate fellows (*n* = 4), implementation partners (*n* = 3), collaborators (*n* = 2), one community member, and one external partner familiar with OHRECA. Representation across thematic areas was nearly balanced.

OHRECA has achieved several milestones that contributed to strengthening One Health research capacity in Africa. These milestones reflect progress across multiple domains, including scientific productivity, human resources, laboratory infrastructure, networks, and community engagement. An overview of key milestones over time is presented in Fig. S1.

### 4.1 Advancing locally led science

**Fig. 2:**
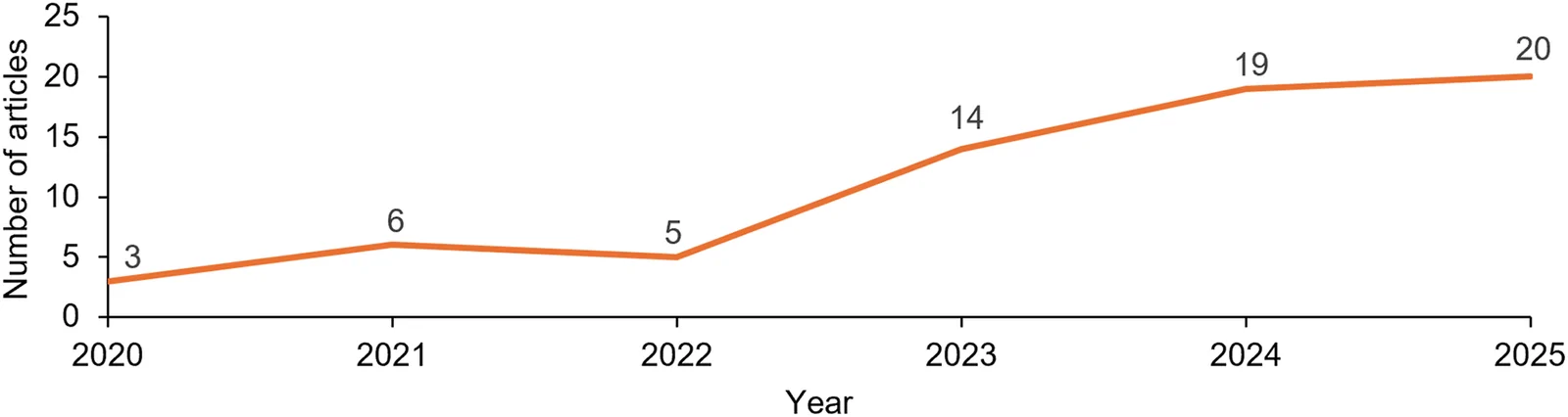
Number of peer-reviewed articles from One Health Research, Education, and Outreach Centre in Africa from 2020 to 2025.

**Fig. 3:**
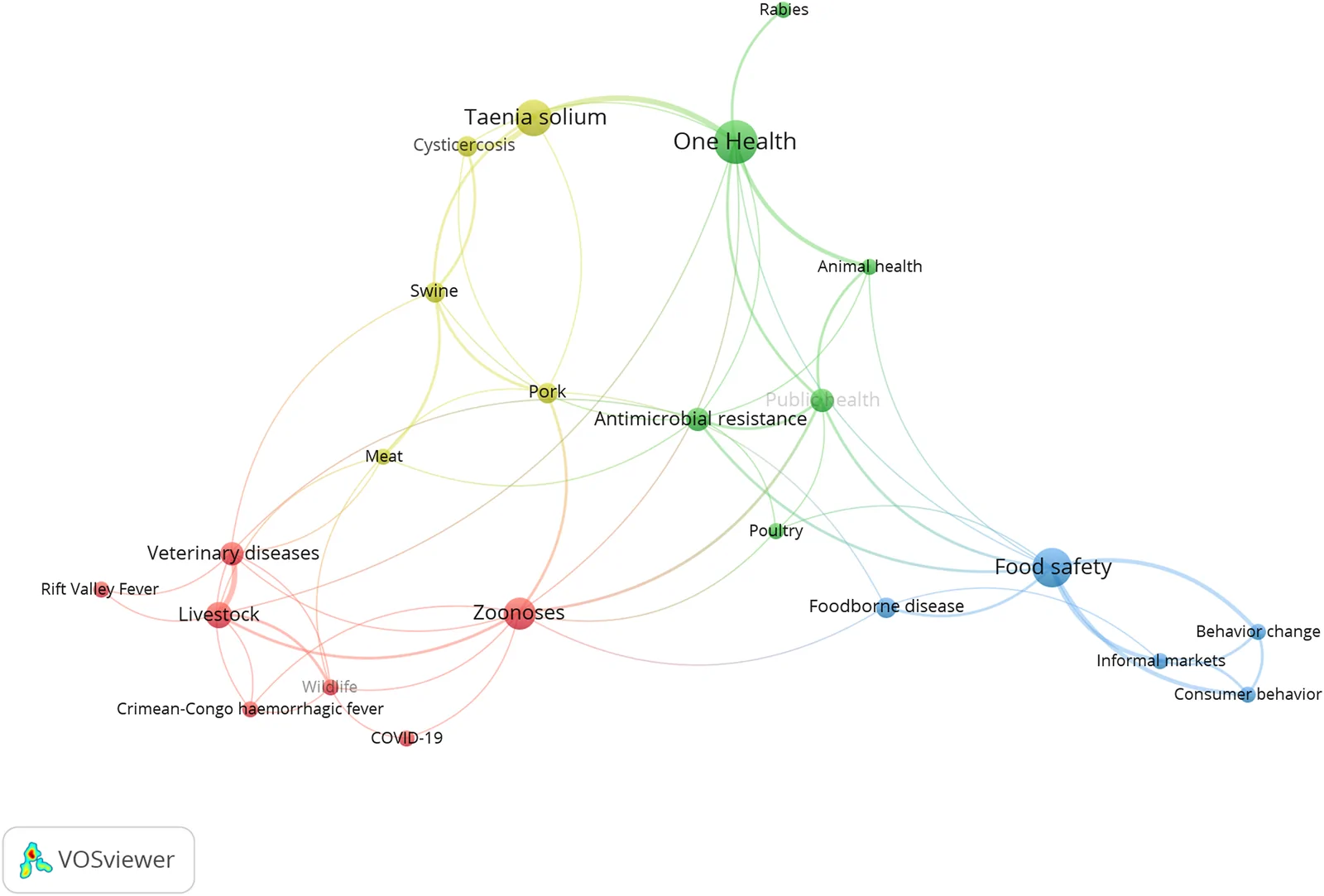
Research topics covered by OHRECA. A co-occurrence analysis of authors' keywords (minimum of three co-occurrences) found in peer-reviewed articles published between 2020 and 2025 (*n* = 67) was performed using VOSviewer (version 1.6.20). Each node represents a keyword, with node size reflecting frequency of occurrence and link thickness indicating co-occurrence strength. Clusters are primarily defined by thematic areas, with methods and study populations appearing as smaller, connected nodes within these clusters. Note that VOSviewer automatically adjusts label visibility and shading to reduce overlap and improve readability.

From the bibliometric analysis, OHRECA made substantial scientific contributions with 67 peer-reviewed publications (as of August 2025) (Fig. 2). OHRECA generated evidence on neglected zoonoses (e.g. *Taenia solium,* rabies) [18,19], emerging infectious diseases (e.g. Crimean-Congo hemorrhagic fever, Rift Valley fever) [20,21], food safety [22], and AMR [23]—primarily using livestock as an entry point, and increasingly wildlife (Fig. 3). Participants noted that OHRECA expanded ILRI's portfolio into the environmental dimensions of One Health. Some work also crossed disciplines, for example by exploring the intersection of AMR and food safety [24], as well as tackled multiple issues within a single thematic area, such as Rift Valley fever and brucellosis under zoonoses.

OHRECA's scientific outputs not only advanced pathogen-specific and interdisciplinary understanding but also strengthened Africa-led authorship. Over 80% of publications (56 of 67) were first authored by graduate fellows or researchers based in Africa, demonstrating strong local leadership and supporting emerging scientific careers.

OHRECA outputs received high citations (Table S2) and Altmetric scores (Table S3), suggesting strong visibility and attention, with the potential for research uptake. OHRECA also actively disseminated its work through videos, reports, and blogs, reaching thousands of people across diverse audiences. Notably, a rabies awareness video received nearly one million views [25], while a news article in *The Conversation*—highlighting findings from a flagship report on the risks of consuming wild meat—was viewed over 50,000 times, as well as republished by at least four media outlets [26,27].

These efforts helped to mobilize new funding, strengthening the sustainability of results. For instance, within CGIAR, scientists highlighted how OHRECA has shaped One Health work under the new CGIAR Science Program “Sustainable Animal and Aquatic Foods” (2025–2030) [28]. Externally, its work informed a new five-year initiative (GBP 2.3 million) targeting *T. solium* Uganda. OHRECA's contributions also positioned ILRI to become a World Organisation for Animal Health (WOAH) “Collaborating Centre for One Health” in 2025, which is expected to open more collaboration opportunities.

Generating evidence is necessary but insufficient on its own to influence policy; continuous political engagement and tailored communication strategies are also needed. Although it is early to assess the policy impacts of OHRECA's locally generated data, several short-term outcomes indicate progress toward future influence. At the county level in Machakos, Kenya, partners reported OHRECA's research informed recommendations for planning rabies prevention activities. At the national level, OHRECA's risk maps have informed revisions to Kenya's national Rift Valley fever contingency plan and human brucellosis testing guidelines. Globally, OHRECA has contributed to shaping strategies for AMR mitigation reflected in the United Nations General Assembly Political Declaration [29].

OHRECA also strengthened pathways for research to inform policy and practice. Graduate fellows reported presenting locally and nationally to knowledge users, and internationally at conferences. Moreover, supervisors served on high-level working and advisory groups that shape decisions and guidelines, including—amongst others—Lancet One Health Commission, World Health Organization (WHO) Guidance Development Group on Traditional Food Markets, Joint FAO/WHO Expert Meetings on Microbiological Risk Assessment (JEMRA), African Union Food Safety Agency, and Global Leaders Group on AMR.

### 4.2 Human resources capacity strengthening

From the project monitoring data, OHRECA trained 34 graduate fellows (65% PhD, 35% MSc), with gender parity (50% women) representing a large, coordinated investments in long-term One Health capacity development. Fellows were fully or partially funded to pursue advanced degrees, reported gaining technical, interdisciplinary, cross-sectoral, and professional competencies through an integrated model of seminar series, mentorship, peer networks, and international placements. Many graduates shared they are now embedded in government and research institutions, where they continue to apply One Health approaches in their professional roles.

Importantly, OHRECA's selection of fellows was strategic: several were already employed in public institutions and returned to their roles with enhanced One Health capacity. For fellows, working in interdisciplinary and cross-sectoral teams added immense value. Furthermore, research outcomes would have been more limited had they remained within disciplinary silos. Many also valued opportunities beyond evidence generation, including engaging directly in intervention and policy initiatives, bridging the gap between research and application.

According to partners, fellows also enhanced scientific productivity and program implementation while maintaining cost-effectiveness. At the same time, partnerships were central to outcomes. For example, collaboration with the University of Burundi enabled laboratory-based research that would otherwise have been infeasible for one fellow, demonstrating how partnerships can overcome capacity and infrastructure constraints.

OHRECA's reach extended beyond its fellowship cohort. Short courses and one-off trainings were delivered to students in multiple countries, including training veterinarians on responsible antimicrobial use in Uganda and Kenya, and veterinary student training programs in Malawi. Partners shared how these broader engagements helped institutionalize One Health thinking within existing training efforts, ensuring that capacity building efforts will continue to scale beyond OHRECA's lifespan.

### 4.3 Laboratory infrastructure

The COVID-19 pandemic in 2020 served as a pivotal moment for OHRECA to demonstrate the importance of partnerships. When the donor asked OHRECA how it could support pandemic containment efforts, OHRECA mobilized scientists across disciplines within and beyond ILRI. According to scientists, this rapid coordination contributed to the establishment of a genomics platform for severe acute respiratory syndrome coronavirus 2 (SARS-CoV-2) variant monitoring and the accreditation of ILRI's biosciences laboratory for human COVID-19 testing, marking a milestone in collaboration between animal and human health research systems.

These efforts resulted in immediate and lasting outcomes. In the short term, scientists highlighted how OHRECA contributed to national COVID-19 surveillance and testing capacity in Kenya as well as fostered continental collaboration on tracking emerging variants. The platform has since been repurposed for other high-threat pathogens, including Crimean-Congo hemorrhagic fever virus, demonstrating how One Health infrastructures such as dual-use laboratories and their teams can pivot during health crises. The key lesson is that sustained partnerships, and not one-off collaborations, are essential for rapid response and for strengthening One Health institutionalization.

### 4.4 Strengthening networks

OHRECA reportedly built on previous investments, including United States Agency for International Development (USAID)-supported efforts to establish and strengthen One Health platforms, thereby avoiding duplication and amplifying impact. For example, OHRECA scientists worked extensively with existing One Health platforms, supporting organizational capacity through One Health assessments, Theory of Change workshops, and South–South exchanges. Although it is too early to assess the long-term outcomes of these efforts, early signals suggest that enhanced capacity could lead to strengthened national roadmaps for One Health implementation.

Much of this work took place in West Africa where ILRI previously had limited presence compared to East Africa. From program monitoring data, in 2023, OHRECA convened 29 representatives from One Health platforms in Mali, Burkina Faso, Senegal, and Côte d’Ivoire for a regional training designed to promote systems thinking, coordination, and South–South exchanges. The workshop introduced participants to the core One Health competencies and guided each country team in developing tailored Theory of Change, with follow-up engagements to support operationalization. This expansion into West Africa demonstrates OHRECA's potential as a continental bridge builder, but highlights the need for deeper institutional partnerships to ensure continuity.

Partners emphasized how OHRECA also directly contributed to establishing One Health platforms where none previously existed, such as in Malawi. Furthermore, OHRECA engaged regionally with the Economic Community of West African States One Health coordination body, informing the development of their regional One Health strategy. A key mechanism behind these outcomes was OHRECA's role as a convener—bringing together experiences and lessons from other contexts and connecting One Health platforms to regional and global initiatives.

Its expertise was highly sought after: Vétérinaires Sans Frontières engaged OHRECA scientists to provide training on One Health assessment tools, while the Food and Agriculture Organization of the United Nations (FAO) invited OHRECA to lead the Rift Valley fever chapter in its new surveillance manual. Partners such as Global Alliance for Improved Nutrition (GAIN) begun applying the “three-stool approach” to food safety, an approach first promoted through ILRI research [30]. Through these engagements, OHRECA helped shape research priorities and strategies to address them.

**Fig. 4:**
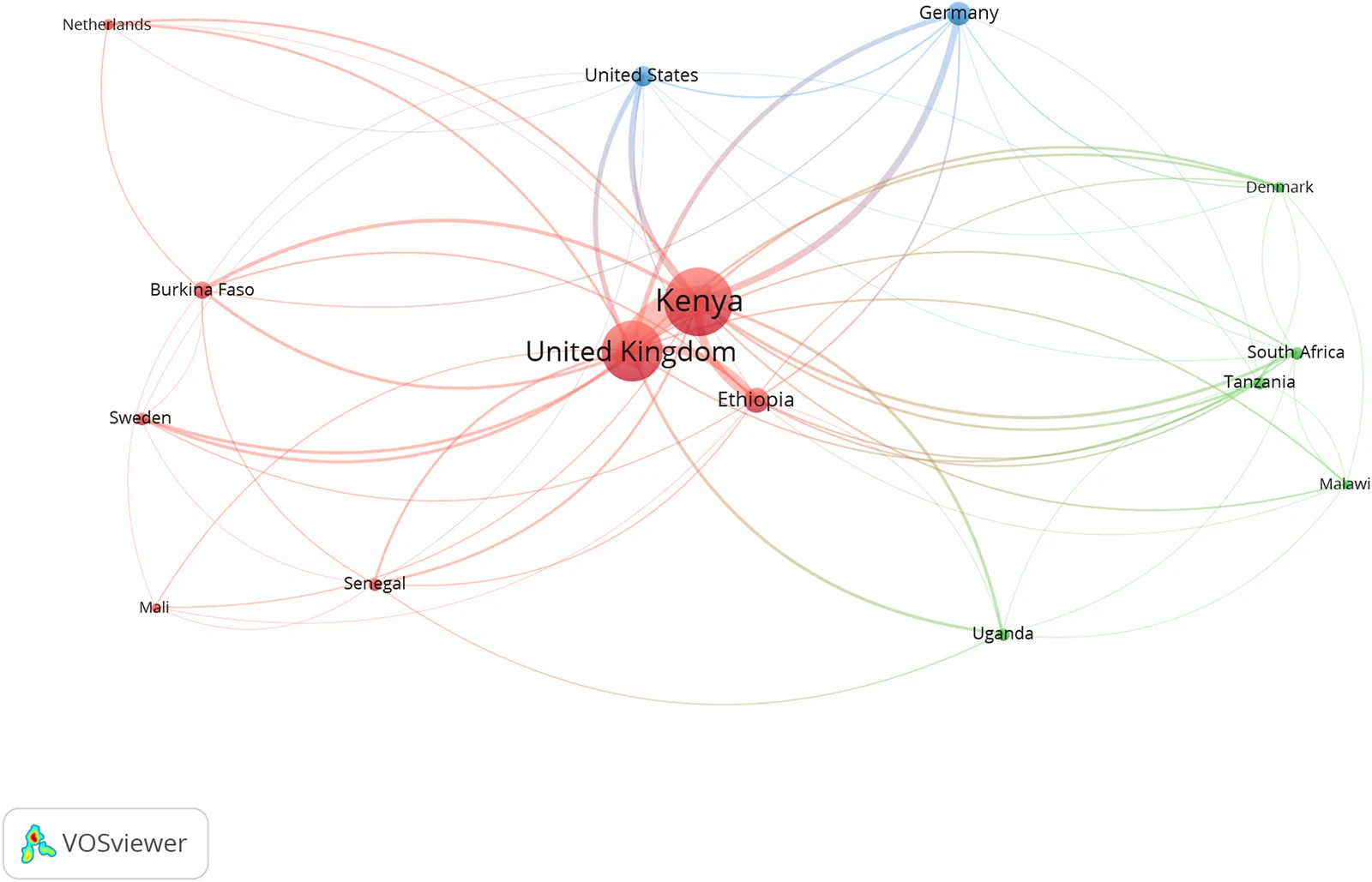
Cross-Africa collaborations built by OHRECA. A co-occurrence analysis of authors' country affiliations (minimum of three co-occurrences) found in peer-reviewed articles published between 2020 and 2025 (*n* = 67) was performed using VOSviewer (version 1.6.20). Each node represents a country, and the node size reflects the frequency of its co-authorship across publications. Link thickness indicates the strength of co-authorship between countries, based on the number of shared publications.

Collectively, these activities have deepened research collaboration across the continent. OHRECA scientists regularly co-authored publications with researchers from at least nine African countries, demonstrating a growing network of scientific exchange and mutual learning (Fig. 4). Moreover, OHRECA's research excellence enhanced the credibility of its partners. A notable example is OHRECA's contribution to the GAIN-led EatSafe project in Ethiopia and Nigeria, where its technical expertise improved the scientific rigor of collaborative studies [31].

### 4.5 Changes on the ground

OHRECA's research-for-development efforts included trainings that supported health behavior change. Program monitoring data, supported by interview accounts, documented outcomes across several initiatives in Kenya—from rabies control in Machakos, to disease surveillance in Isiolo, and food safety training in Nairobi. These three case studies illustrate how OHRECA's research-for-development approach translated knowledge into action at community level.

In Machakos County, OHRECA led a large-scale rabies control program that combined scientific research, vaccination campaigns, and community engagement. In partnership with government (County Department of Veterinary Services, County Department of Public Health) and implementation partners, the team conducted baseline studies to characterize dog populations, optimize vaccine logistics, and ultimately vaccinated 136,000 dogs with coverage in high-risk wards reaching 60% by the end of the program. While this effort fell short of the 70% coverage threshold required to interrupt rabies transmission at the population level, partners mentioned it provided key insights for coordinated rabies control in this county and more broadly. Notably, community engagement was central to the campaign's success. A total of 120 animal health service providers and 318 community health promoters (CHPs) were trained on rabies transmission, prevention, and community education techniques. Partners indicated CHPs now go on to integrate rabies messaging into routine household visits which sustains public awareness.

In Isiolo County, Kenya, OHRECA contributed to strengthened disease surveillance systems through community-based capacity building. Implemented in partnership with the Isiolo One Health platform, the initiative trained 120 community members—including livestock keepers, community health volunteers, and local leaders—on disease recognition, reporting procedures, and risk communication. Through the post-training evaluation, the team found improvements in both knowledge and attitudes toward disease surveillance and response. The practical impact became evident during a Rift Valley fever outbreak in June 2024. Trained community members participated in syndromic surveillance, promptly reporting suspected cases and animal abortions to the County Department of Veterinary Services. Partners reported a substantial increase in community-initiated reports during the outbreak period which supported vaccination efforts. This approach has since been replicated in other counties, including Marsabit, contributing to a growing model for decentralized, community-driven disease surveillance. Despite high participation, sustaining vaccination rates required local government ownership and recurrent funding, a key lesson for program design.

OHRECA also helped to advance food safety practices in Kenya's informal meat markets through a combination of research, hands-on training, and public awareness initiatives. Working closely with local authorities, market managers, and implementation partners, the team conducted intensive capacity building sessions for 200 butchers, focusing on practical demonstrations of hygiene practices and use of personal protective equipment. While these efforts reportedly improved butchers' knowledge, microbiological assessments showed limited reductions in contamination, underscoring the importance of not only training but also infrastructure and regulatory support. Beyond market-level interventions, OHRECA expanded its outreach through targeted communication campaigns. Collaborating with five local radio stations, OHRECA broadcasted messages on meat hygiene and food safety, tailored to reach diverse audiences including youth, elders, and ethnic communities. Though not yet evaluated, scientists mentioned it is likely that messages reached tens of thousands of consumers who can then advocate for safer food practices among vendors.

Together, these experiences show that One Health capacity is strengthened not just by research or training, but by co-implementation with communities and authorities.

### 4.6 Challenges and lessons learned

OHRECA faced challenges related to scope, planning and evaluation, and team composition, many of which were shaped by external factors beyond OHRECA's control. These experiences, however, generate valuable lessons for future One Health efforts.

According to scientists and partners, the scope of OHRECA was at times both overly broad and, paradoxically, not ambitious enough. While the program covered an expansive agenda across research, capacity building, and policy engagement, often stretching available resources, it did not always go far enough in translating evidence into action. For example, although the national One Health capacity assessments identified key gaps, the subsequent implementation work, while present through certain One Health platform activities, was comparatively limited in scope. More targeted activities here could have enhanced their practical value and long-term impact.

In terms of planning and evaluation, scientists mentioned strong leadership from the coordinating unit and regular internal meetings helped move center activities along. However, working across themes proved challenging, which meant missed opportunities to address multiple, interconnected issues at once. The shift from theme-based activities to cross-cutting action areas improved integration, but only to some extent, as funding structures remained tied to themes. This highlights the need for institutional changes in how research funding is allocated. Furthermore, a shared implementation and evaluation framework was missing—this could have helped to align activities and monitor progress. It is worth noting OHRECA was launched just before the COVID-19 pandemic, and as such, OHRECA had limited opportunities for in-person meetings—an extended inception phase might have helped to support planning for integration.

Recruiting fellows already embedded within government agencies proved effective for building lasting institutional capacity, with several now driving One Health initiatives within their organizations. However, enrolling government staff as students was sometimes challenging as competing job responsibilities limited their availability. On the other side, some fellows mentioned more time from supervisors would have been appreciated. A student-supervisor agreement could have enhanced this experience. Partnership effectiveness also varied across contexts—for instance, collaborations with implementation partners were stronger in some countries more than others—highlighting the importance of careful partner selection, clear expectations, and active oversight.

Political and institutional contexts substantially shaped OHRECA's implementation. At the county level, progress was sometimes hampered by inconsistent cost-sharing arrangements and frequent staff turnover. However, newly recruited officers were typically enthusiastic, offering fresh opportunities for engagement. Most interactions also occurred with technical staff rather than decision-makers, which limited OHRECA's ability to influence policy directions. Nonetheless, the relationships developed could facilitate stronger access to leaders over time. At the national level, political sensitivities—such as governments' reluctance to declare disease outbreaks due to potential trade repercussions—often constrained their engagement. To mitigate these challenges, OHRECA played a convening role, fostering cross-sector coordination that helped to ease tensions. Engaging with regional bodies like East African Community also helped to reduce national-level hesitations.

This evaluation has several limitations. First, although conducted by an external evaluator, the evaluation involved close collaboration with program implementers, which may have introduced potential biases in data collection, interpretation, and reporting. In addition, qualitative findings were based on detailed interview notes rather than verbatim transcripts, and coding was conducted by a single researcher without formal double coding. Although member checking enhanced the accuracy of the findings, these methodological choices may have limited the depth of the qualitative analysis. Second, the absence of a standardized implementation and/or evaluation framework limited comparability across sites, although it helped adapt activities to diverse contexts and identify context-specific pathways to outcomes. Third, as this realist evaluation focused on understanding how and why outcomes occurred rather than assessing effectiveness, baseline or comparator data were not available for many outcomes, limiting causal attribution. Furthermore, some observed outcomes may reflect broader influences beyond OHRECA alone. Finally, the initiative was situated in a strong institutional setting, which may limit generalizability to contexts with more limited institutional capacity. However, evaluating projects at different stages of development, and implemented with partners of varying capacity, provides insights across a range of real-world conditions.

## 5 Conclusion

Over six years (2020–2025), OHRECA made notable contributions to One Health research capacity in Africa, generating locally led evidence, developing skilled human resources, enhancing laboratory infrastructure, building networks, and driving change on the ground. Some outcomes are likely to sustain beyond OHRECA's funding cycle through established alumni networks, One Health embedded in university curricula, and strengthened national and regional platforms. At the same time, challenges related to scope, planning and evaluation, and team composition underscore the need for balance (e.g. between assessments and interventions), shared implementation frameworks, and clearly defined partner expectations. By identifying the mechanisms and contextual conditions associated with program outcomes—including learning-by-doing, partnerships, long-term investment, and institutional continuity—this study provides timely insights into supporting locally led One Health capacity across diverse African research settings, particularly as global efforts to strengthen One Health systems, pandemic preparedness, and health security continue to grow.

## CRediT authorship contribution statement

**Steven Lâm:** Writing – original draft, Conceptualization. **Bernard Bett:** Writing – review & editing, Supervision, Conceptualization. **Arshnee Moodley:** Writing – review & editing. **Florence Mutua:** Writing – review & editing. **Lian Thomas:** Writing – review & editing. **Delia Grace:** Writing – review & editing, Conceptualization.

## Consent for publication

All authors consent to publish.

## Availability of data and materials

The study generated qualitative interview and bibliometric data. Qualitative interview data are not publicly available due to confidentiality considerations. Bibliometric data were derived from a publicly available source (CGSpace) and are also available upon request.

## Funding

This work was supported by the 10.13039/501100006456Federal Ministry for Economic Cooperation and Development (10.13039/501100006456BMZ), Germany. It was also part of the CGIAR Sustainable Animal and Aquatic Foods Program, which was supported by contributors to the 10.13039/501100015815CGIAR Trust Fund (https://www.cgiar.org/funders). CGIAR is a global research partnership for a food-secure future dedicated to transforming food, land, and water systems in a climate crisis.

## Competing interests

Bernard Bett and Delia Grace serve as the editorial board members of *Science in One Health*. They are not involved in the peer review, editorial assessment, or any other process that could affect the handling of this manuscript. The authors have no other competing interest to disclose.
