# Supplementary material for: Six years of One Health action in Africa: evaluating outcomes, mechanisms, and lessons from OHRECA (2020–2025)
Source: Sci One Health. 2026 Jul 24;5:100166. doi: 10.1016/j.soh.2026.100166 (PMC13544079; doi:10.1016/j.soh.2026.100166)
Supplement: Supplementary file 1 [file mmc1.docx]

**Fig. S1.** Overview of key OHRECA milestones. An interactive version is available at: https://tours.dobiison.com/Enterprise/ILRI_OHRECA.

**Table S1. Context, mechanism, outcome configurations.**

| **Outcome at-a-glance** | **Outcome elaborated** |
| --- | --- |
| **Neglected zoonotic diseases** | |
| Evidence | |
| Responding to government priorities | Uganda government’s interest in cysticercosis vaccines improved **(outcome)** through a wiliness to pay study **(mechanism)** in a context where government commissioned the study **(context) (interviewee #11)** |
| Reaching hard-to-reach areas | Work on pork cysticercosis expanded to a hard-to-reach area in northern Uganda **(outcome)** by adapting earlier value chain tools from central Uganda **(mechanism)**, building on previous neglected zoonoses research **(context) (11)** |
| Setting up groundwork | New collaborations were established in northern Uganda **(outcome)** by initial joint work in value chain assessments **(mechanism)**, in a region where ILRI previously had limited reach **(context) (11)** |
| Capacity building | |
| Intersectoral coordination | Coordination between veterinary and public health departments in Kenya improved **(outcome)** through the establishment of quarterly meetings that facilitated joint planning around rabies control **(mechanism), in contexts where counties can host meetings and daily** allowances were provided **(context) (6, 19)** |
| Interventions | |
| Improving community interest | Strong community support and high turnout for vaccinations in Kenya **(outcome)** by engaging trusted community health promoters to mobilize dog owners and address their concerns **(mechanism)** in rural areas where veterinary services are limited and dogs are essential for security and herding **(context) (16)** |
| Building disease response preparedness | Community disease reporters improved rapid outbreak detection and response in Kenya **(outcome)** after receiving RVF training on recognition, reporting, and risk communication **(mechanism)** in high-risk communities **(context) (1)** |
| Addressing rabies | Vaccinated 136,000 dogs (~30% coverage) and identified cost and logistical needs to reach the 70% threshold **(outcome)** by conducting operational research and coordinating a rollout with community sensitization **(mechanism)**, in a setting where Kenya had a national rabies strategy and partnerships in place but limited resources for effective implementation **(context) (4)**  The research team provided scientific input the Machakos County government to guide the design and scaling of mass rabies vaccination efforts **(outcome),** achieved by conducting studies on dog population size and cost-effectiveness **(mechanism) (5)**  Reduced bite-related risks and eased the financial burden of expensive post-bite treatments in Kenya **(outcome)** by providing preventive vaccination for dogs **(mechanism)** in communities where treatment costs around 5,000 KES for five injections, considered unaffordable for many families **(context) (16)**  Residents in Machakos County in Kenya began to recognize the value of dogs **(outcome)** after conducting rabies vaccination campaigns **(mechanism),** in a context where dogs were initially considered low-value animals compared to livestock **(context)** (19) |
| Influencing implementation partners | The rabies vaccination strategy developed by OHRECA was applied to other settings **(outcome) given the adaptability of the approach (mechanism) in a context where the partner does similar work (context) (13)** |
| Policy | |
| New funding | Secured funding for a 5-year RCT (SUSTAIN project) to test interventions for T. solium in Uganda **(outcome)** by generating epidemiological data, conducting contextual analyses, and engaging stakeholders **(mechanism)** (5)  Have new funding for deworming and vaccine trials for pork tapeworm in Uganda **(outcome)** by leveraging evidence generated from earlier value chain mapping, willingness-to-pay studies, and gender analyses **(mechanism)** (11) |
| Improved disease reporting | Developed and integrated a rabies reporting module into Kenya’s national surveillance system **(outcome)** by leveraging the institutional positioning and training of an OHRECA fellow **(mechanism)**, in a context where reporting systems were weak **(context) (5)**  **Rabies case reporting in Kenya improved by 40% (outcome), made possible by enhanced reporting channels (mechanism) (13)** |
| **Emerging infectious diseases** | |
| Evidence | |
| Understanding CCHF | New evidence on CCHF in Burkina Faso **(outcome)** by conducting studies using a One Health approach **(mechanism)** in a setting where localized data on CCHF had been scarce **(context) (15)** |
| Informing disease response | OHRECA improved RVF preparedness in Kenya (**outcome**) by generating more accurate, county-level risk maps (**mechanism**) (18) |
| Capacity building | |
| NA | NA |
| Interventions | |
| COVID-19 response | OHRECA helped to establish a genomics platform **(outcome)** by mobilizing ILRI expertise, seeking human diagnostic accreditation, and leading sequencing efforts **(mechanism)** during a period when Kenya needed to expand testing capacity **(context) (12)**  OHRECA strengthened national capacity in Kenya and contributed to global insights on COVID-19 variants **(outcome)** by providing laboratory and scientific expertise **(mechanism)** during the pandemic **(Context) (1)** |
| Disease surveillance support | Reporting of suspected outbreaks such as RVF and PPR increased in Uganda while response times decreased **(outcome),** achieved through sensitizing farmers, providing targeted training for district officers, and strengthening links between district staff and epidemiology units **(mechanism) (13)** |
| Improving community interest | Communities in Burkina Faso actively engaged in discussions about zoonotic diseases and CCHF **(outcome)**, through early involvement of communities as data collectors as well as training community health workers (**mechanism**), even though malaria and dengue were perceived as more pressing **(context) (15)** |
| Policy | |
| Informing plans | OHRECA helped to update Kenya’s RVF contingency plan **(outcome)** by providing technical expertise, updated case definitions, and risk maps **(mechanism)** in response to recurring outbreaks and outdated planning tools **(context) (1)** |
| New funding | OHRECA secured €8 million in BMZ funding which later increased by €7 million during COVID-19 **(outcome)** through a proposal to support COVID-19 testing **(mechanism)**, following the success of ILRI-led BUILD project which had built trust of BMZ **(context) (12)** |
| Future funding potential | African Union partners invited the team on a proposal on CCHF **(outcome)** facilitated by CCHF research completed **(mechanism) (15)** |
| **Food Safety** | |
| Evidence | |
| Improvements in food safety knowledge | Observable but modest improvements in food safety knowledge and practices were achieved in Ethiopia **(outcome)** through vendor training and consumer engagement **(mechanism),** during a 5–6 month EatSafe intervention in informal markets with limited regulatory oversight **(context) (10)** |
| Complementary studies for a collaborator | Additional activities to added to EatSafe program **(outcome)** thanks to ILRI-led rapid hazards studies and complementary program evaluation in Ethiopia and Nigeria **(mechanism) (10)** |
| Strengthened research excellence for a collaborator | More rigorous findings and higher-quality publications as part of EatSafe **(outcome)** because of ILRI field research expertise **(mechanism)**, addressing GAIN’s limited internal capacity for research-intensive tasks **(context) (10)** |
| Setting up groundwork | Foundation created for future food safety projects in Burundi **(outcome)** achieved by initiating research activities **(mechanism)** in a context with limited prior research in food safety and One Health **(context) (8)**  Improved collaboration between veterinary and public health departments **(outcome)** through joint trainings and coordinated activities **(mechanism)**, in a setting where previously fragmented mandates separated oversight of slaughter (veterinary) and retail meat safety (public health) **(context) (8)**  Established a foundation for other initiatives (e.g. COHESA) to engage in **(outcome)**, through engagements with community and government partners **(mechanism)**, in a context where there was limited past ILRI work **(context) (3)** |
| Capacity building | |
| Improved awareness | **Youth, elders, and different ethnic groups gained awareness of meat hygiene and food safety (outcome) through radio campaigns with 5 local FM radio campaigns targeting different audiences (mechanism) (2, 20)** |
| Interventions | |
| Influencing projects of collaborators | GAIN’s EatSafe food safety interventions became more holistic **(outcome)** by informally adopting ILRI’s “3-legged stool” framing of training, enabling environments, and incentives **(mechanism)**, at a time when GAIN was expanding into food safety in Ethiopia and Nigeria **(context) (2)** |
| Better hygienic practices | Meat vendors in Nairobi gained better awareness of safe food handling practices and showed strong willingness to adopt improved practices **(outcome)** through training sessions and the distribution of personal protective equipment, which fostered buy-in **(mechanism),** in settings where public health officers were present **(context)** (8, 20)  Two hundred butchers were trained, and those adopting these techniques reported higher sales due to cleaner and safer meat presentation **(outcome)**. This shift resulted from practical, hands-on training and visible demonstrations that motivated behavior change **(mechanism)**, within informal market chains where hygiene practices were often inconsistent **(context) (13)**  There were some marginal improvements in hygiene practices and risk perception among meat handlers **(outcome)**. This was made possible by delivering training sessions, providing personal protective equipment, and running radio messaging campaigns **(mechanism)**, despite working in traditional markets where hygiene is poor and the implementing partner had limited capacity and oversight **(context) (2)** |
| Policy |  |
| Influence in global food safety dialogues | EatSafe project findings in Ethiopia and Nigeria gained visibility in global policy spaces **(outcome)** through ILRI’s strategic engagement in platforms like the UN Food Systems Summit and media coverage such as Delia Grace’s World Food Day interview **(mechanism) (10)** |
| Future policy influence | Institutions now have access to a harmonized food safety curriculum, improving consistency in training and qualifications across the region **(outcome)**. OHRECA supported this by benchmarking curricula with IUCEA **(mechanism)**, in response to the varied food safety training across East African institutions **(context) (3)** |
| **AMR** | |
| Evidence | |
| **Advancing understanding of AMR-environment links** | The research improved understanding of AMR dynamics in environmental settings in Kenya **(outcome)**. This insight emerged from the interdisciplinary integration of soil science, microbiology, and One Health approaches **(mechanism)**, in a context where AMR research has historically focused more on human and animal health than environmental pathways **(context) (17)**  Global recognition of the environment as a critical component of AMR was strengthened **(outcome)** through evidence generation and thought leadership in UNEP technical reports and UN policy processes, including the UN General Assembly Political Declaration on AMR **(mechanism)**, in a global discourse that had historically prioritized clinical drivers over environmental **(context) (7)**  OHRECA advanced research on AMR in the environment **(outcome)** by analyzing residues in pig and poultry farms and modeling ecological and human health risks **(mechanism)** in a field that is less studied compared to human and animal health **(context)** (9)  Awareness of the neglected environmental dimension of AMR was raised among policymakers and researchers **(outcome)** through presentations delivered during World AMR Awareness Week and at international conferences **(mechanism) (17)** |
| **Laying the groundwork** | Preliminary evidence suggests potential for influencing antimicrobial use at the farmer level in Malawi **(outcome)** through mechanisms such as piloting context-sensitive stewardship messages, aligning farmer incentives, and improving delivery pathways for AMR education **(mechanism) (7)** |
| Cross-sectoral collaboration | Collaboration between animal health and environmental health sectors improved **(outcome)** by deliberately engaging UNEP and integrating environmental scientists into OHRECA activities and advisory structures **(mechanism)**, in a context where AMR discussions had traditionally been underrepresented in environmental discussions **(context)** (7) |
| Capacity building | |
| Veterinary curriculum | A national training curriculum was developed and is ready for scale-up to improve antimicrobial use in Uganda and Kenya **(outcome)**. The curriculum was co-developed with government partners **(mechanism)**, addressing existing knowledge gaps among veterinary medicine distributors **(context) (14)**  Collaboration with VSF Germany supported the development of training curricula for veterinarians in Uganda and Kenya **(outcome)** through joint design on evidence-based modules **(mechanism)** in communities with limited access to structured training on prudent antimicrobial use **(context)** (9) |
| Interventions | |
| Better practices | Seventy percent of the 1,200 targeted farmers adopted better husbandry practices, reducing disease risk, and 160 veterinarians were trained to support responsible antimicrobial use in Uganda **(outcome)**. These results were driven by on-farm training sessions that linked farmers directly with veterinarians **(mechanism) (13)**  Poultry farmers significantly increased vaccine uptake and reduced antibiotic use, with a noticeable decline in over-the-counter antimicrobial sales in Kenya **(outcome)**. These changes were supported by training programs that promoted a “vet-diagnostic-first” approach, ensuring expert guidance before antibiotic purchases **(mechanism) (13)** |
| Groundwork | Preliminary evidence suggests potential for influencing antimicrobial use at the farmer level in Malawi **(outcome)** through mechanisms such as piloting context-sensitive stewardship messages, aligning farmer incentives, and improving delivery pathways for AMR education **(mechanism) (7)** |
| Policy | |
| Support to government NAP | The Senegalese government invited ILRI to help develop and implement its 2023–2027 AMR NAP **(outcome)** as a result of credibility of OHRECA work **(mechanism)**, building on ongoing collaborations with the OHP **(context) (4)**  A foundation for influencing updates to national AMR action plans in Kenya was established **(outcome)** through research examining antimicrobial use, treatment guidelines, and regulatory and stewardship frameworks **(mechanism) (7)** |
| Future influence | OHRECA could inform antimicrobial stewardship strategies in Kenya and Malawi **(outcome)** by systematically mapping existing policies and identifying gaps to guide more coordinated and actionable approaches **(mechanism) (9)** |
| **Cross-cutting** | |
| Evidence | |
| Knowledge generation | High-quality evidence, such as RVF risk maps, AMR studies, and peer-reviewed publications, was produced to advance scientific understanding and inform advocacy and decision-making **(outcome)**. This was achieved by building on USAID-funded tools and lessons, including OH assessments and joint risk assessments **(mechanism) (18)** |
| Recognition by for expertise | FAO invited OHRECA to lead the RVF chapter in its new surveillance manual **(outcome)**, recognizing the team’s technical leadership in risk mapping and preparedness **(mechanism),** amid growing international interest in improving zoonotic disease surveillance **(context) (1)**  Africa Risk Capacity sought OHRECA’s support to build a RVF dashboard **(outcome)**, leveraging risk mapping work **(mechanism)** in response to regional needs for tools to detect disease risks **(context) (1)**  VSF Senegal adopted the NEOH tool for project evaluation and benefited from peer learning opportunities **(outcome)** after inviting ILRI to provide technical training and support **(mechanism)**, driven by OHRECA’s reputation for using the tool **(context) (4)**  ILRI’s reputation as a technical partner in One Health grew in West Africa **(outcome)** through field research, policy engagement, and capacity development **(mechanism)**, in a context where ILRI had a relatively limited presence compared to East Africa **(context)** (4)  Invitations to contribute to expert panels, such as the Lancet One Health Commission and CODEX **(outcome)**, achieved by leadership in neglected zoonoses and foodborne disease with high-quality publications **(mechanism) (5)** |
| Capacity building | |
| South–South knowledge and capacity exchange | OHRECA enabled knowledge exchange between project countries **(outcome)** by strategic country selection aimed at building African-led One Health capacity while learning from platforms like Afrique One **(mechanism) (12)** |
| One Health platforms | Uganda’s One Health platform was revitalized **(outcome)** through multi-stakeholder meetings that strengthened cross-sector coordination **(mechanism) (13)**  Evidence-based recommendations for capacity-building efforts, aligned with national priorities, were produced **(outcome)** after OHRECA conducted assessments to identify and validate One Health capacity gaps **(mechanism)**, in a context where many National One Health Platforms lacked implementation support **(context) (3)**  National One Health representatives from West African countries were engaged in cross-learning **(outcome)** facilitated by regional workshops **(mechanism) i**n a context where structured collaboration had been limited **(context) (15)**  National OH platforms in West Africa strengthened their ability to move from strategy to implementation and monitoring **(outcome)** through structured capacity building, including multi-country workshops and Theory of Change exercises informed by baseline NEOH assessments **(mechanism)**, in a context where platforms existed but lacked clear implementation roadmaps **(context) (4)** |
| Graduate fellow training | PhD students, including those from the BUILD Uganda program, were supported and local research teams were strengthened **(outcome)** through targeted funding and mentorship that enabled locally grounded research **(mechanism)** **(11)**  OHRECA trained interdisciplinary student cohorts with social science, biomedical, and applied research skills **(outcome)** through investments in both hard and soft skills, peer learning networks like WhatsApp groups, regular engagement with theme leads and communications staff, and a recruitment model that drew from multiple disciplines and often government institutions **(mechanism) (12)**  Fellows in Burkina Faso gained hands-on experience applying One Health approaches and bridging theory with practice **(outcome)** through practical fieldwork and intentionally designed interdisciplinary collaboration **(mechanism) (15)**  Students built advanced research skills and generated evidence to inform future interventions **(outcome)**. This was achieved by funding and mentoring six graduate students (two PhDs and four MScs) at the University of Nairobi on topics such as Brucellosis and Q fever **(mechanism) (14)**  Graduates became more employable, with some moving into roles in government and international organizations **(outcome)**. This was made possible through mentorship, technical training, and exposure to international networks and platforms provided by OHRECA **(mechanism) (3)**  Built a cadre of alumni now embedded in government and international organizations, such as Mission Rabies, driving long-term systems change **(outcome)** through recruitment and mentorship of high-potential graduate fellows with a focus on applied research, systems thinking, and partnership building **(mechanism) (5)**  The interviewee advanced their expertise and career trajectory **(outcome)** through doctoral training in Sweden and ILRI, interdisciplinary One Health research exposure, and participation in international research networks **(mechanism)** (8)  Research capacity and the pipeline of trained food safety professionals expanded **(outcome)** by involving graduate fellows and junior researchers in EatSafe in Ethiopia data collection and analysis **(mechanism)**, filling a gap in academic hosting and skills transfer within GAIN **(context) (10)**  A new cohort of internationally trained but locally embedded researchers is strengthening human resource capacity for food safety and One Health in LMICs **(outcome)**. This was achieved by securing placements in Sweden and Germany and providing strategic support to students **(mechanism)**, in a context where students from LMICs have high demand for degrees from HIC institutions **(context) (2)**  Researchers, particularly veterinarians, gained practical One Health knowledge to better address zoonotic and food safety challenges in Malawi **(outcome)**. This was achieved through OHRECA-supported training and research opportunities for early-career professionals **(mechanism) (3)**  The soil researcher in Kenya strengthened collaboration skills and gained interdisciplinary perspectives **(outcome)** by accessing structured training in grant writing, scientific writing, and literature review, as well as interdisciplinary mentoring **(mechanism)** **(17)**  OHRECA built capacity of future AMR leaders **(outcome)** by training multiple PhD students and embedding them in applied research and international networks **(mechanism)** in LMIC settings where skilled professionals are critical but institutional support are often limited **(context) (9)** |
| Interventions | |
| NA | NA |
| Policy | |
| Influence on policy dialogues | OHRECA contributed to policy dialogues at national and regional levels **(outcome)** by maintaining effective communication, leveraging senior advisory members, and embedding students in government agencies **(mechanism) (12)**  ILRI/OHRECA became a trusted contributor to regional strategy development **(outcome)** by leveraging repeated engagement, trusted relationships, and strong technical expertise **(mechanism)**, building on past USAID initiatives and ongoing involvement in West Africa **(context) (4)** |

**Table S2.** Most cited OHRECA articles.

| Author | Title | Journal | Number of citations |
| --- | --- | --- | --- |
| Mwatondo et al. (2023) | A global analysis of One Health Networks and the proliferation of One Health collaborations | *Lancet* | 123 |
| Gilbert et al. (2021) | Review: Mitigating the risks posed by intensification in livestock production: The examples of antimicrobial resistance and zoonoses | *Animal* | 71 |
| Fasina et al. (2021) | The One Health landscape in sub-Saharan African countries | *One Health* | 51 |

**Table S3.** OHRECA articles with highest altmetric scores.

| Author | Title | Journal | Altmetric score |
| --- | --- | --- | --- |
| Chepyatich et al. (2023) | Seroprevalence of *Toxoplasma gondii* in slaughtered pigs in Kiambu, Kenya | *Zoonotic Disease* | 98 |
| Mwatondo et al. (2023) | A global analysis of One Health Networks and the proliferation of One Health collaborations | *Lancet* | 59 |
| Patel et al. (2023) | Assessing disease risk perceptions of wild meat in savanna borderland settlements in Kenya and Tanzania | *Frontiers in Ecology and Evolution* | 51 |
